# Touching at a Distance: Digital Intimacies, Haptic Platforms, and the Ethics of Consent

**DOI:** 10.1007/s11948-021-00338-1

**Published:** 2021-09-21

**Authors:** Madelaine Ley, Nathan Rambukkana

**Affiliations:** 1 Ethics/Philosophy Section, Department of Values, Technology and Innovation, Faculty of Technology, Management and Policy, Delft University of Technology, Delft, Netherlands; 2 Communication Studies, Faculty of Arts, Wilfrid Laurier University, Waterloo, Canada

**Keywords:** Ethics of technology, Haptics, Digital intimacy, Platform studies, Communication technologies, Teledildonics

## Abstract

The last decade has seen rise in technologies that allow humans to send and receive intimate touch across long distances. Drawing together platform studies, digital intimacy studies, phenomenology of touch, and ethics of technology, we argue that these new haptic communication devices require specific ethical consideration of consent. The paper describes several technologies, including Kiiroo teledildonics, the Kissenger, the Apple Watch, and Hey Bracelet, highlighting how the sense of touch is used in marketing to evoke a feeling of connection within the digital sphere. We then discuss the ambiguity of skin-to-skin touch and how it is further complicated in digital touch by remediation through platforms, companies, developers, manufacturers, cloud storage sites, the collection and use of data, research, satellites, and the internet. Lastly, we raise concerns about how consent of data collection and physical consent between users will be determined, draw on examples in virtual reality and sex-robotics, and ultimately arguing for further interdisciplinary research into this area.

## Introduction

Touch is an important mode of communication for humans; we have the ability to offer support, love, disdain, or discomfort through small but meaningful contact. Until recently, it would seem absurd to say you could physically touch someone in another room or country; emerging haptic technologies, however, make this possible—albeit in a new way. With the rise of one-person households (Semega et al., 2019; Snell, 2017; Yeung & Cheung, 2015), increased rates of loneliness that some label an “epidemic” (Gerst-Emerson & Jayawardhana, 2015; Leigh-Hunt et al., 2017; Luo et al., 2012), and most recently the isolation introduced by COVID-19, the market for digital touch may be growing. As interpersonal touch enters the digital realm to augment other modes of online communication there are new ethical considerations, and in this paper we specifically consider the role of physical and digital consent in the use of new haptic technologies.

The following paper takes a multidisciplinary approach, drawing on literature from digital intimacy studies (Andreassen et al., 2017; Dobson et al., 2018; Miguel, 2018; Rambukkana, 2015a, 2015b), phenomenology and post-phenomenology (Al-Saji, 2010; Liberati, 2017; MacLaren, 2014), and ethics of technology (van de Poel, 2013; van Wynsberghe & Robbins, 2014; Nissenbaum, 2001). In drawing these fields of thought together, we are able to recognize the myriad ways in which contemporary intimacy is and will be shaped by the development of haptic technologies, and discern some of the unique ethical consent concerns that arise with the emergence, development, and futures of digital touch.

## Digital Intimacy and Platform Studies

Digital intimacy studies unpacks the varied ways humans are intimate in our digitalized world, often—and increasingly—via platforms. As a framing, “digital intimacies” connects two often disparate fields of research. While digital culture studies frequently explores the interconnections, interactivity, and proximities that such technologies afford (Rheingold, 1993; Odzer, 1997; O’Riordan & Phillips, 2007; Baym, 2010; Paasonen, 2011), only rarely has this work been considered in relation to the study of “intimacies” specifically (McGlotten, 2007; Rambukkana, 2015a, 2015b; Rambukkana & Gautier, 2017; Attwood, 2017). Similarly, while critical intimacy studies addresses the impact of media on the intimate public sphere broadly (Berlant, 1997), critical intimacy studies explorations of digital platforms remain rare (Miguel, 2018). As Rambukkana (2015b) has argued, the critical conjunction of “digital intimacies” connects these complementary fields.

This paper mobilizes the emerging fields of both digital intimacies and platform studies. Critical intimacy studies provides an important framework for the growing sociocultural phenomenon of digital intimacy, which research has shown (Penley & Ross, 1991; O’Riordan & Phillips, 2007; Paasonen, 2011; Hasinoff, 2015; Philips, 2016; Baym, 2018; Attwood, 2017) drives transformative change in how people develop and express intimate relationships using technology.

Scholars have variously defined intimacy. Berlant (1998, p. 282) considers intimate relationships as “the close connections that matter, and on which we depend for living.” Bersani and Phillips (2008, p. vii) define it as “the source and medium of personal development.” While “intimacy” has been discussed by religious and philosophical thinkers for millennia, the formulation favoured in critical intimacy studies emerged from queer theory. Queer theory (Jagose, 1996) has made significant contributions to our theoretical understanding of intimacy in modern contexts. In particular, it extended its study beyond kinship and sexuality studies to incorporate problematics on all scales, from internal dynamics of personalities and interests, to interpersonal and group dynamics, to macro-social organizations.^1^ Viewed as a whole, intimacy studies have explored the problematics of identities (Bersani & Phillips, 2008; Butler, 1990, 1993, 2004), publics and counterpublics (Berlant, 1997; Calhoun, 1992; Fraser, 1992; Habermas, 1989; Warner, 2002), and societal privilege (Combahee River Collective, 1977; Clark, 2000; Heldke & O’Connor, 2004; Rambukkana, 2015a). Most pertinent to this study, intimacies are relevant to friendships (Bickmore, 1998), networks (Zappavigna, 2011), kinship (Haraway, 1992; Harrison & Marsden, 2004), and sexualities (McGlotten, 2007; Rambukkana, 2015a).

The majority of digital media research addresses forms of intimacies, if not always in those words. This includes problematics such as cyberlibertarianism (Barlow, 1996; Bey, 1991; Dyson et al., 1994), virtual communities (Rheingold, 1993; Barney, 2003; Feenberg & Barney, 2004), cybersex (Odzer, 1997), online publics (Kolko, 2003), women’s online space (Shade, 2003), queer online identities (O’Riordan & Phillips, 2007), digital pornography (Paasonen, 2011), and sexting panics (Hasinoff, 2015). However, this research has only recently been framed as platform studies.

Montfort and Bogost (2009, p. 2) define a platform as “a computing system of any sort upon which further computing development can be done. It can be implemented entirely in hardware, entirely in software (which runs on any of several hardware platforms), or in some combination of the two.” Moreover, platform studies may include other peripheral texts, from the examination of underlying code, to terms of service, to packaging and advertising materials, even to the ownership structures of companies. A growing field, platform studies has recently expanded from video game systems (Bogost & Montfort, 2009; Jones & Thiruvathukal, 2012), to explore broader issues, such as algorithmic culture (Gillespie, 2014; Crawford, 2016) and social media platforms (Burgess & Matamoros-Fernández, 2016; Langlois & Elmer, 2013). However, this field is still in its infancy, with particularly acute research gaps in haptic interfaces; by focusing on digital intimacy and haptic platforms, this paper tries to address one of these gaps—especially in relation to intimate communication and questions of ethics and consent. This paper shifts the focus from connections made through visual, audio, and linguistic means to the haptic intimacies that can occur with the use of technology.

## Haptic Technologies as Platforms for Intimacy

Many contemporary technologies involve haptic interaction between the user and the physical device. A text message accompanied with a vibration, for example, is a communication method that incorporates haptics and can cause an affective, even intimate, response in the recipient. When feeling a phone buzz, a person may respond with a jolting sensation of surprise, excitement, or dread depending on their context and/or expectations. However, the sense of touch in this example is a tool to indexically link someone to the primary mode of communication, which in this case is either visual (text or photo messages) or audio (voice message or phone call). This paper focuses instead on technologies that use the sense of touch as the primary mode of communication and strive towards mutual interaction between people across a distance and (usually) in real-time. While other senses, like vision or hearing, help facilitate the feeling of connection through touch, they play a more supportive role here. Below we provide a description of several such haptic platforms that facilitate a range of intimate relationships, drawing attention to the language used in their marketing to highlight how the sense of touch supposedly presents digitally. Later, we discuss two further technologies implicated with touch: VR and sex robotics, to flesh out a full discussion of the implications of digital touch consent.

### Teledildonics

Howard Rheingold coined the term *teledildonics* in the 1990s, presciently predicting a future where one could use sex toys online to engage with others despite physical distance:

You probably will *not* use erotic telepresence technology in order to have sexual experiences with machines. Thirty years from now, when portable telediddlers become ubiquitous, people will use them to have sexual experiences with other *people*, at a distance, in combinations and configurations undreamed of by precybernetic voluptuaries (1991, p. 345, emphasis in original)

Current teledildonic companies (such as Kiiroo) and companies that offer teledildonics as part of their product ranges (such as We-Vibe) are the manifestation of Rheingold’s prophecy. For example, Kiiroo produces fleshlights and vibrators that can be connected and synchronized online, so that couples can experience a sensation similar to sexual intercourse without skin-to-skin contact. Kiiroo has several devices available for purchase and while some pairings include only unidirectional control, where just one person can affect the force, speed, or pattern of their partner’s device, other combinations allow for the possibility of mutual control. These sets can be found on their webpage under the “Couples” category, where an advertisement reads: “The Kiiroo Couple Set 2 was designed to ease the distance and close the gap, because who wouldn’t like to feel their lover’s intimate touch when they are away?” (Kiiroo, 2021). An advertisement for another set reads, “The two-way connection enables you and your partner to share your pleasure from any distance. The built-in touch-sensitive technology allows for bi-directional control of connected devices so either of you can drive the action” (Kiiroo, 2021).

Kiiroo’s marketing focuses on closing distances, as well as mutual pleasure and agency. The language carefully makes clear that the couple using their technology is not having “sex” as it is traditionally understood, but rather are “mimicking intercourse in real-time” (Kiiroo, 2021). Indeed, the possibilities for a digital sexual encounter via Kiiroo are limited to the capabilities of the technology. For example, when using Kiiroo, intimate touch is centred around the genital area. The person using the vibrator has the ability to be more creative because they can move the device anywhere on the body, shifting pressure, and changing speed. The person using the fleshlight, however, is limited to a repetitive up-and-down motion that can shift in speed, stroke length, or pattern only. While the options, compared to skin-to-skin sexual interaction, are reduced, unique possibilities also emerge with the use of this technology—the most obvious being new ways to be intimate and feeling sexually connected across a distance. But there are also emergent perils, such as deception about who might be on the other end of the device, hacking the control feed, or even the illegal distribution of recorded intimate sessions—some of these potentially rising to “rape by deception” (Rambukkana & Gauthier, 2017; Sparrow & Karas, 2020).

### Kissenger

The Lovotics website explains that the Kissenger is a device that focuses on intimate touch around the lips and makes it possible to digitally kiss someone through an attachment on a smartphone or a stand-alone device. Kissenger has three applications: human-to-human, human-to-robot, or human-to-virtual character. For our purposes we focus on the first, which is described as aiming to:

Bridge the physical gap between two intimately connected individuals. Kissenger plays the mediating role in the kiss interaction by imitating and recreating the lip movement of both users in real time using two digitally connected artificial lips. (Loveotics, n.d.)

Kissenger recreates the pressure of a person’s mouth on their partner’s corresponding device in real time, meaning that the constant attunement that occurs when kissing lip-to-lip might be (to some degree) experienced. As with Kiiroo’s and We-Vibe’s teledildonic sets, the couple’s experience is largely formed by the shape, texture, and affordances of the technology. Unlike a lip-to-lip kiss, for example, there is no moisture, warmth, or possibility of tongue involvement. Yet, people using Kissenger may become used to the digitally mediated interaction and experience it like a kiss or something that signifies a kiss. Again, with the development of this technology a new kind of digital intimacy emerges and the experience of distance between loved-ones shifts.

### Apple Watch

Apple has long traded in haptic metaphor, apt for a company that pays such attention to tactile detail; from physical user interface (UI) design to the niceties of packaging, Apple products are always crafted with pleasing touch experiences in mind. The iPhone, which was a paradigm shift in both touch screen telephony and mobile internet, was also framed as a way to let “music lovers ‘touch’ their music” (Apple, 2007).

The iPad was initially marketed as a “magical and revolutionary” way to “physically interact with applications and content” (Apple, 2010), one often short-handed to literally “touching the internet” (e.g., Gonyea, 2010). In 2014–15 they started flirting even more deeply with touch, introducing their Taptic Engine with the MacBook Pro Force Touch trackpad, allowing haptic feedback (Apple, 2015a); and 3D Touch with the 6S iPhone line, which added “new ways to navigate and experience iPhone by sensing pressure to enable new gestures” (Apple, 2015b). But with the 2014 Apple Watch, their entrée into the wearables market, Apple showed a commitment to making haptics not only utilitarian, but intimate. The Apple Watch “blurs the boundary between physical object and user interface” (Apple, 2014), with an always-touching UI that mobilizes multiple haptic technologies: “Force Touch, a technology that senses the difference between a tap and a press”; “the Taptic Engine […] that […] discreetly enable[s] an entirely new vocabulary of alerts and notifications you can […] feel”; and “Digital Touch, [that allows you to send] something as personal as your own heartbeat” (Apple, 2014). Its blend of passive touch with the ability to “send” touch messages to other Apple Watch users marks this as arguably the next-gen haptic technology with the widest user base.

### Hey Bracelet and Hey Touch

The focus of Hey is to create a haptic communication device that enables non-sexual intimate touch. The company produces two devices, Hey Bracelet and Hey Touch, which are described as both filling the haptic gap in digital communication and “adding a completely new dimension to relationships” (Hey, 2021). Hey’s marketing focuses on the capability touch has to communicate support or affection to a loved one, and positions its haptic technology as an opportunity to continue this over long distances. When using Hey Bracelets, two people have the sleek device around their wrists and when one person lightly squeezes theirs, the other person’s “produces a gentle squeeze” letting you “send a ‘real’ human touch across distance” (Hey, 2021). There is no vibration or buzz for bystanders to see or hear, so the interaction may remain private between the two people involved.

The Hey Touch is the latest development by Hey and offers more types of touch beyond the bracelet’s simple squeeze. Hey Touch is a small stylish square that one can wear as a necklace or tuck in their pocket. Connected through an application, one can send up to 200 types of touch to another user (Hey, 2021). Like Apple, the Hey Touch is trying to integrate their technology into everyday communication. The device can be linked to messages or pictures, and can be a part of a group chat: “Thanks to the integrated multicoloured LED, it’s clear who’s sending the touch. Say ‘I miss you’ or good luck’. Let loved ones know you’re thinking of them, have fun with a group of friends or use it for all kinds of other occasions” (Hey, 2021). While the Hey Touch can be used to send a haptic message, it can also be used to enhance other digital communication already occurring.

## The Ambiguity of Touch

Marketing of digital touch platforms, as shown above, mobilizes the affective quality of touch. Discussions on digital touch have unique difficulties in finding clear language, as the sense of touch is frequently used metaphorically. When affected by a loving gesture or piece of art, we say: “I was so touched”; after a mental breakthrough, we exclaim: “Then it hit me!”; in conveying something beyond rational thought, we describe: “I feel that…”; and in moments of disconnect, we admit: “I’m just not grasping it.” While the context in which such utterances arise may vary, the use of touch is similarly called upon to help articulate an experience or lack of affect or connection.

This mixing of metaphorical and literal is intentionally used for marketing—as with the play of the word “platform” (Gillespie, 2010), a doubling that makes “touching platforms” a particularly layered site of analysis. The ambiguity appeals to potential users’ emotions, while also suggesting that the device will allow them to literally touch their loved ones. While digital touch is made possible through developers’ research on aspects such as force and pressure, a user would not experience a squeeze on the Hey Bracelet in this highly quantified way. The squeeze would likely give the user a sensation that is physical *and* emotional, largely shaped by the context, relationship, and intimate history between the two people, intermixed with and framed by the meanings and discourses mobilised by the platform makers in their packaging, marketing, instruction materials, etc.

Beyond this affective tangling, consider also the fundamental paradox in the very idea of touching long-distance. Historically it would be nonsensical to suggest that you could squeeze someone’s hand from a distance. But by drawing on the metaphorical meaning of touch, i.e., being emotionally affected, and articulating this together with new haptic technologies, touching long-distance becomes both conceivable and possible—while remaining phenomenologically distinct from immediate skin-to-skin contact.

Digital touch technology differs from immediate physical contact in that it incorporates both distance as well as hardware and software intermediations. Yet, the ambiguous nature of old fashioned skin-to-skin touch remains. Feminist phenomenologist Alia Al-Saji draws on Husserl’s conception of touch and bodily awareness, developing the notion of *sensings*, a level of the body as a surface that is totally in touch with the world, making for an intimate relationship of “proximity and reciprocity” (2010, p. 19). According to this conception, the body is both being-touched and participating in the affective sense of being-touched (p. 23). Thus, the act of being-touched is not simply passive because the affect it inspires also requires activity. Al-Saji uses the passive–active dynamic to suggest that there is a blurring of self and other in inter-personal touch (p. 18). Though the body seems to move spontaneously, it does so in active-passivity and responds to the “affective pull” of the world it inhabits (p. 25). Bodies are receptive to their situation and move accordingly; a person’s hand will jerk away when surprised or will lighten its pressure when perceiving tenderness on another. When two sentient beings touch, a co-sensing occurs: when a person reaches out and touches another’s arm her hand is actively touching but also adapting to possibilities that allow the other to be touched. For example, she may have to reach up to caress the other’s face or move slowly in order to not scare him or her away. Connection in touch, then, is complex and involves blurry boundaries—one is both touching and touched, pursuing and responding, and therefore, as bodily awareness is interconnected with haptic experience, others can play a role in shaping a person’s embodied subjectivity.

Kym Maclaren provides further insight into the intersubjective ambiguity in touch by drawing on the Merleau-Pontian idea that the body is both subject and object (2014). Maclaren clarifies that this should not be understood as a dichotomy; instead she suggests thinking about the body as both sensible and sentient, where these “are essentially intertwined: our being-sentient is inseparable from our being-sensible” (2014, p. 98). Touch is a cooperative movement connecting the subject and the world. The touching agent is guided by the being or thing they intend to touch. Maclaren gives the following example: “To feel the softness of the fuzz on a baby’s head, one must not pat vigorously, but rather keep a certain distance and move one’s hand gently back and forth” (p. 99). Although one person may seem more active in the physical connection, touch involves a mutual activity of responding and being affected by the other. This physical connection between two beings is never quite static and each body provides both input *and* responses, thus neither being can be identified as wholly active or wholly passive.

The concepts of attunement and the blurring of active and passive are still present in this emergent digital touch connectivity. Despite what marketers would have people believe, the experience of using technologically mediated touch differs fundamentally from immediate bodily contact. It takes on a new form, shaped according to the technological capabilities of the device, the priorities of the developers, and ultimately by user-experience (UX). Instead of attuning immediately to the flesh of another, a user attunes to the technology and how the other’s body is perceived, represented, or remediated over a distance.

From a phenomenological standpoint, the devices become inhabited by the user’s bodies thereby changing the world around them (Liberati, 2017). At first, using the digital touch technologies will be clumsy and the devices will be noticeable; the remediation aspect weighs heavily at this stage. For example, as intimate as the Apple Watch’s Digital Touch affordances are, their UI is clunky and feeling the Taptic Engine beat a rhythm on your wrist is markedly different from a tap on your shoulder or hand resting on the small of your back. However, with more use, and more finely grained design elements, the devices could become incorporated into the person’s body, not just as an unnoticed extension, but part of the body schema itself. Devices like Hey Bracelet or the Apple Watch could become so integrated by the body as to become mundane ways of communicating—much as the tactile vibration of cell phones or game controllers, once novel, has already become—opening up new ways of being with others in the world. Technologies like the Kissenger and teledildonics like Kiiroo could transcend feeling like a simulation of touch and “feel” more like a real thing, if not *the exact same kind* of real thing. This fuzzy complexity, this intersubjective mutuality, the role of technology as intermediary and co-actor, and this potential to *become* something more fundamentally authentic than a marketing gimmick are also why consent issues loom large, as we turn to in the next section.

## Ethics, Consent, and Haptic Platforms

Shifting from a strictly phenomenological lens, which focuses on the experience of subjects using haptic technology, we distinguish digital touch from immediate touch in order to highlight that there is a network involved in transmitting a touch message across distances. Digital touch involves platforms, companies, developers, manufacturers, cloud storage sites, the collection and use of data, research, satellites, and the internet. What may seem private and intimate in fact involves a huge expanse of activity undergone with unknown, even unknowable, partners. As Carey Jewitt et al. point out, “[d]igital touch does not only raise questions of trust in the relationship between people but also in the reliability, security and safety of the machines and systems that mediate touch” (2020, p. 116). This foregrounds the issue of how consent is determined in the privacy policies and data collection of the companies. While people may be unlikely to divulge their sexual preferences and activities to a stranger in person, the concern for privacy often wanes online. Pornography sites, for example, account for 30% of web traffic despite how sites can leak user data, such as gender/sexual identity and sexual interests (Maris et al., 2019). Contributing to users’ uncritical engagement with online privacy is how user agreements are often so long and complicated that people typically scroll through without reading and click “agree.” While consent is usually technically stated, we can hardly call it “informed.” The intimate information that can be collected through digital touch devices should be treated with appropriate sensitivity. Kiiroo, for example, states that they collect minimal data because the information is sensitive, but not all companies adhere to this ethos.

The information collected by haptic technologies also helps to create a comprehensive documentation of a person’s identity and body—this not only includes their intimate practices and desires, but can also include their body shape, temperature, texture, and heartbeat. Such metadata is crucial to platform capitalism with its increasing reliance on user data (Srnicek, 2016, p. 39). This is even more pertinent when such “data-driven intimacy” (p. 279) is articulated to sexuality, what Flore and Pienaar (2020) term a “sexuotechnical-assemblage” (p. 279). A case in point is the March 2017 class action suit against Canadian company Standard Innovation, for failing to inform its customers that their wireless We-Vibe sex toy was quietly collecting user data such as “time and date of use, the user-selected vibration intensity level and pattern and the temperature of the device” (Perkel, 2017, n.p.), resulting in a 5-million-dollar settlement (Perkel, 2017).

Beyond data consent, haptic platforms also raise questions about how physical touch can or should be determined. The #metoo movement has spurred macro-level cultural conversations about the nature of sexual consent, ones that need careful consideration when translated to digital haptics. Feminist movements have pushed for a shift from the no-model of consent, where only a verbal “no” draws the boundary between acceptable and unacceptable, to a yes-model, where absence of an on-going “yes” indicates lack of consent (Anderson, 2005). Under this model, consent is not the default or settled with a once-given simple “yes” in either non-sexual or sexual touch (Anderson, 2005); it is ongoing, sometimes supported with verbal exchange, sometimes with signs of pleasure or sighs of comfort. It is revoked with a “no,” but also with a shift-away of the eyes, or tense body. Yet, the yes-model of consent is not without its own troubling ambiguities. In response to the “Yes Means Yes” campaigns adopted in colleges across the United States, critics argue that men continually misread women’s body language (Anderson, 2005) or avoid checking-in with their partner to avoid an explicit refusal (Jozkowski, 2015), and people tend to find that voicing consent explicitly can feel transactional and awkward (Willis et al., 2019). A feminist ethics of consent is not black and white, often shifting according to myriad factors including the type of relationship, context, mood, and timing. Accounting for this complexity will be a challenge for those who are developing and using technologies that mediate intimate touch between two or more people.

These ambiguities of consent continue into the digital realm. Even in the absence of more advanced haptic interaction, where one could feel another’s entire body, there are many possibilities for miscommunication and boundary violation. For example, one could increase the speed of a lover’s vibrator without sensing what, in close proximity, would usually be telling body language signals to slow down. Full communication through touch is only possible with attunement to the whole of the contextual elements. Like homographs, which can only be interpreted correctly within a full sentence, certain bodily sensations are only understood within the fullness of the body. Tension, for example, can alternately indicate ecstasy or discomfort. This distinction may be lost over digital devices. In addition, the world of digital haptics might lack scripts, conventions, mores, or laws. In the examples of virtual reality (VR) and sex robotics, we can see how such emergent technologies strain notions of consent.

### Virtual Reality

Jordan Belamire describes a virtual groping encounter within a multiplayer HTC Vive VR game *QuiVR*:

In between a wave of zombies and demons to shoot down, I was hanging out next to BigBro442, waiting for our next attack.

Suddenly, BigBro442’s disembodied helmet faced me dead-on. His floating hand approached my body, and he started to virtually rub my chest. “Stop!” I cried. I must have laughed from the embarrassment and the ridiculousness of the situation. Women, after all, are supposed to be cool, and take any form of sexual harassment with a laugh. But I still told him to stop.

This goaded him on, and even when I turned away from him, he chased me around, making grabbing and pinching motions near my chest. Emboldened, he even shoved his hand toward my virtual crotch and began rubbing.

There I was, being virtually groped in a snowy fortress with my brother-in-law and husband watching.

As it progressed, my joking comments toward BigBro442 turned angrier, and were peppered with frustrated obscenities. At first, my brother-in-law and husband laughed along with me—all they could see was the flat computer screen version of the groping. Outside the total immersion of the *QuiVr* world, this must have looked pretty funny, and definitely not real.

Remember that little digression I told you about how the hundred-foot drop looked so convincing? Yeah. Guess what. The virtual groping *feels just as real*. Of course, you’re not physically being touched, just like you’re not actually one hundred feet off the ground, but it’s still scary as hell. (Belamire, 2016; emphasis added)

This is obviously a clear violation, a lack of consent that was communicated in multiple ways: verbally, with gestures, with moving away. But even with more everyday encounters, due to the lost ability to communicate nuanced needs and desires through feeling each other’s bodies, strong verbal communication would need to increase to ensure ongoing and enthusiastic consent. Relying on verbal communication alone is not enough, however, and reaching the fullness of the yes-model of consent requires further technological advancement, such as a haptic body suit that could transduce nuanced touch—with the Teslasuit as one example of tech moving in that direction (Teslasuit, 2019).

### Sex Robotics

A limit case pertinent to these issues could be intimate relationships with robotic beings as “digital others” (Levy, 2007; Liberati, 2018; Viik, 2020). Levy (2020) notes that critics of sex robots—such as Richardson (2022)—argue that a robot, as object, could never consent to sex (p. 191). While this is an as-yet-unresolved ethical question that would require knowing the shape of future technology to fully determine, he also notes how a parallel question in light of the #metoo inflected discussion of consent, might be “How can a robot determine, with any degree of certainty, whether or not a proximate human wants or at least consents to sex?” (Levy, 2020, p. 191, 197). Questions of what behaviours are acceptable from the robot and who is responsible in the event of consent violations from a sex robot are also raised (Levy, 2020, p. 191). Pinning his analysis to the notion that advanced robotics could use “sexual scripts” to understand—or, problematically, even *infer* or “*optimize”*—consent (Levy, 2020, 192), his analysis underlines how multiple senses play a role in the negotiation of consent, from verbal and visual elements to touch and body language cues (Levy, 2020, 194). More worrying than anything, however, is Levy’s suggestion that sexual consent violations committed by an autonomous robot—in other words, robotic sexual assault—should be treated as an accident only and remedied by vehicles such as insurance rather than legal proceedings (Levy, 2020, 198).

Other issues of consent can continue from the skin-to-skin to the digital world. An abusive or controlling partner or family member could incessantly send touches through an Apple Watch or Hey Bracelet, thereby extending physical control across distances. In some ways, however, digital touch technology might provide the possibility of an empowering sense of control. Being able to quickly shut the devices down or move it away from the body easily removes one from the other’s touch. Of course, in many relationships where there is abuse and unsafe power dynamics, taking a drastic stance of control like shutting off a digital touch device could feel nearly impossible. Since the effects of corporeal miscommunication or transgression can be damaging, mitigating its continuance in the digital world is important to ensure users’ safety. The need for consent considerations is clear with respect to haptic platforms and, as demonstrated above, is both emergent in effects and unclear with respect to scope. But thinking through present and future technologies where haptic consent is being wrangled with can help to unpack the stakes of these issues.

## Conclusion: Negotiating Haptic Futures

With new touch technologies, it is now possible to have our touch dispersed. However, it is not possible to touch in the exact way one would in person—this freedom is stifled by the mediating technology. With digital touch it is not merely two or more people adapting and attuning to each other’s bodily situations but rather an entire infrastructure constructing the design and distribution of digital touch. This very infrastructure might be mobilized to facilitate digital and physical consent. A growing movement in the philosophy of technology argues that most decisions made in a technology’s lifespan, from conception of to dissemination and use, result from implicit or explicit prioritization of particular values (Van de Poel & Kroes, 2014; Friedman & Nissenbaum, 1996; Nissenbaum, 2001; van Wynsberghe & Robbins, 2014). Values are defined broadly as “what is important to people in their lives” (Friedman & Hendry, 2019 p. 23), which depends “substantively on the interests and desires of human beings within the cultural milieu” (Friedman et al., 2006 p. 2). Privacy, safety, and efficiency are examples of values commonly prioritized in technologies. We argue that physical consent be added to the list when it comes to haptic technologies. Further research might include taking a Value-Sensitive Design (Friedman & Hendry, 2019) or Design for Values approach (van den Hoven et al., 2015) to consent in haptic technologies to see how it can be translated into design ethics. As we have shown, consent is complex and may be difficult to achieve through the mediation of technology, but design features might also enable it, such as the addition of a disconnect button.

Clearly, when haptic technology interfaces with aspects of human intimacy, design needs to be proactive about ethical considerations, and not just because of the danger of bad press or lawsuits from lapses or hacks. In the game design field, for example, Jess Marcotte (2018) argues that games should be designed with intersectional feminist principles in mind. This extends to games incorporating touch, as Marcotte elaborates:

*In Tune*, which I co-designed, is an example of a game that was developed around intersectional feminist principles. *In Tune* is a game where players are asked to negotiate consent separately from sexual intimacy […]. Players are asked to perform a series of sustained poses, negotiating who will do what to whom, whether the pose needs to be modified, and whether they will perform the pose at all. The game positions consent as an intersectional feminist issue that affects our day-to-day interactions with others and that requires active, ongoing engagement rather than the binary, one-time giving and receiving of consent in sexually intimate contexts. One of the poses asks players to negotiate touching each other’s heads, which finds echoes in a game like *Hair Nah*, which is about (white) people touching a black woman’s hair without her permission [...]. Games like *Hair Nah* and *In Tune* demonstrate some of the ways that one can design intersectionally. (Marcotte, 2018)

Developing and incorporating a feminist ethics of touch, for example, would require that a device had the capacity to mediate or facilitate mutually created consent between users—even if one of the users is itself an artifact, as with sex robots. Linking humanities scholars working on consent with the teams building these technologies is crucial if digital touch is to stay up-to-date with the evolution of consent in the non-digital sphere.

Finally, as communication will adapt to the technologies being used, so too may the nature of consent. For example, if there was an auto-disconnect button it might soon take the place of a safe word, or a user may be able to individualize settings so she only receives touch from certain people at certain times. The specific configurations of consent will be shaped by the unique configurations of users, technologies, and sociotechnical conventions, but tech developers need to provide the options to do so. Future academic work might look beyond affordances and discursive framing of haptic devices to investigate the empirical aspect of how these technologies are thought out by designers, debated by law and policy makers, and experienced by users. One of the limitations of the current work is that it asks many questions, yet answers few. If digital intimacies are shaped by the collision of connection and technologies, how do we map and account for the new terrain of intimacy those encounters create? Generating some potential answers through empirical means would empower further inclusion of interdisciplinary perspectives from ethicists, designers, lawmakers, and users in multiple settings, and could help shape digital touch technologies to avoid extending, in addition to modes of touching, the attendant potential harms of skin-to-skin touch. Similarly, designers should continue to use visions of possibilities and abuses from speculative fiction, and actual anecdotes of the same from critical journalism, to help nuance their products and tweak their affordances. While we do not argue that digital touch is the same as its analog analogue, we establish above it is a real and material experience. As such, we need to take haptic platforms seriously, and thinking about consent is a key piece of that.

## Footnotes

1 Nationality and patriotism are significant forms of intimacy, for example: borders and boundaries of nations create insides and outsides, forms of belonging, and national narratives (Berlant, 1997). Zoning in cities strongly determines intimacies, defining or breaking up neighbourhoods, lifestyles, economies—such as zoning that shutters LGBTQ businesses (Warner, 1999). Intimacies can emerge from fandoms (Bury, 2005; Rambukkana, 2007), from musical subcultures (Thornton, 1995; Baym, 2010, 2018), from indiscretions (Kipnis, 2003; Wasserman, 2015), or from transactions (Feenberg & Bakardjieva, 2004; Zelizer, 2005).
